# Interventions in Small Island Developing States to improve diet, with a focus on the consumption of local, nutritious foods: a systematic review

**DOI:** 10.1136/bmjnph-2021-000410

**Published:** 2022-10-20

**Authors:** Emily Haynes, Eden Augustus, Catherine R Brown, Cornelia Guell, Viliamu Iese, Lili Jia, Karyn Morrissey, Nigel Unwin

**Affiliations:** 1 European Centre for Environment and Human Health, University of Exeter, Exeter, UK; 2 George Alleyne Chronic Disease Research Centre, University of the West Indies, Bridgetown, Barbados; 3 Pacific Centre for Environment and Sustainable Development, University of the South Pacific, Suva, Fiji; 4 Institute for Manufacturing, University of Cambridge, Cambridge, UK; 5 Department of Technology, Management and Economics, Technical University of Denmark, Lyngby, Denmark; 6 MRC Epidemiology Unit, University of Cambridge, Cambridge, UK

**Keywords:** malnutrition, nutrient deficiencies, dietary patterns, metabolic syndrome

## Abstract

**Introduction:**

Food security in Small Island Developing States (SIDS) is an international policy priority. SIDS have high rates of nutrition-related non-communicable diseases, including obesity and type 2 diabetes, micronutrient deficiencies and, in many, persistent childhood stunting. This is associated with an increasing reliance on imported processed food of poor nutritional quality. Calls have been made for strengthening local food systems, resilient to climate change, to increase the consumption of nutritious locally produced food. We aimed to systematically review interventions intended to improve diet in SIDS, and specifically explore whether these interventions applied a local food approach.

**Methods:**

The search strategy was applied to 11 databases, including in health, social science and agriculture. Screening of titles, abstracts and data extraction was undertaken in duplicate. Risk of bias was assessed using Cochrane tools. Narrative synthesis of the results was undertaken. The study protocol was registered (PROSPERO registration number: 2020CRD42020201274).

**Results:**

From 26 062 records, 154 full texts were reviewed and 24 were eligible. Included studies were from the Caribbean, Pacific, Mauritius and Singapore. Five were a randomised study design, one an interrupted time series analysis, eight controlled and ten uncontrolled pre-test and post-test. Nine studies included some aspect of a local food approach. Most interventions (n=15) included nutrition education, with evidence of effectiveness largely limited to those that also included practical skills training, such as vegetable gardening or food preparation. Three studies were considered low risk of bias, with the majority (n=13) of moderate risk.

**Conclusion:**

There is a lack of robust evidence on interventions to improve diet in SIDS. The evidence suggests that multifaceted approaches are likely to be the most effective, and local food approaches may promote effectiveness, through mechanisms of cultural and contextual relevance. Further development and evaluation of interventions is urgently required to increase the comparability of these studies, to help guide policy on improving nutrition in SIDS.

**What is already known on this topic**

- Increasing local nutritious food production is suggested for improving local diets and food security in Small Island Developing States (SIDS).

**What this study adds**

- Multifaceted interventions show greatest promise.
- Local food approaches may promote effectiveness through mechanisms of cultural and contextual relevance.

**How this study might affect research, practice or policy**

- We require high-quality, multifaceted SIDS-based dietary interventions, informed by local cultures and contexts.

## Introduction

Small Island Developing States (SIDS) are disproportionately impacted by the triple burden of malnutrition.^1^ This includes the clinical manifestations of overconsumption and underconsumption of energy and inadequate consumption of micronutrients, namely, overweight and obesity, nutritional deficiencies (such as anaemia in women), childhood stunting and increasing prevalence of non-communicable disease (NCD).^1 2^ The majority of SIDS are located in the Pacific and Caribbean regions, which have some of the highest prevalence of overweight and obesity, and type 2 diabetes, in the world.^3 4^ More than one in five adults are expected to die from an NCD in most SIDS before their 70th birthday.^5^

The major determinant of these challenges is attributed to rapid dietary changes in SIDS, associated with the ‘nutrition transition’.^6^ Over the past three decades, diets within these states and territories have shifted from the consumption of local tubers, roots, fruits and other vegetables towards diets high in saturated fat, added sugar and sodium, particularly from ultraprocessed and processed foods.^6^ These changes are attributed to globalisation, increased access to and availability of imported, non-native and often processed food, and a fall in local agricultural production for local consumption.^5^ Moreover, environmental conditions, extreme weather events becoming more frequent due to climate change, and economic factors such as poor economies of scales, distance to market and limited infrastructure have led to a dependence on food importation which in turn exacerbates food insecurity for many SIDS.^5 7 8^

An increase in sustainable, local food production, particularly the production of diverse, unprocessed or minimally processed foods, that promotes intra-regional trade and links within local communities, is envisaged by the United Nations (UN), Food and Agricultural Organisation (FAO) and SIDS governments as a major component in addressing the triple malnutrition burden.^2 9–11^ It is suggested that improved local food production and an emphasis on nutrition-sensitive value chains may help to offset the severity of these aforementioned shocks to the food system.^1^ For SIDS and similar settings, cross-sectional data suggest that the way food is sourced may impact dietary diversity^12^ and that own food production, through growing a diverse range of crops, may contribute to greater dietary diversity.^13^ However, evidence from interventional research is required to identify the most effective ways of realising this approach.^14^ To our knowledge, this systematic review is the first to assess the effectiveness of interventions in SIDS, aimed at improving diet, and in particular, the first to explore if and how these interventions apply a local food approach. It builds on a broader scoping review on the health and other impacts of community food production in SIDS,^14^ and aims to provide evidence required to guide related practices, programmes and policies in SIDS and other low-income and middle-income countries.

## Methods

The protocol for this review was registered with the International Prospective Register of Systematic Reviews (PROSPERO registration number: 2020CRD42020201274) and reported in accordance with the Preferred Reporting Items for Systematic Reviews and Meta-Analysis (PRISMA) guidance.^15^

Specific objectives of this review were (1) to identify published and grey literature evaluating interventions in SIDS, from any part of the food system, that aim to improve diet or nutrition knowledge; (2) to evaluate the quality of these studies; and (3) to provide narrative and, as appropriate, statistical summaries of the findings, highlighting interventions that aim to improve diet by increased consumption of locally produced foods.

### Eligibility criteria

Inclusion and exclusion criteria are summarised in box 1. A 20-year timeframe was chosen to ensure that included research was relevant in informing future interventions.

Box 1 **Eligibility criteria**

Inclusion criteria

- Interventions implemented and evaluated in one or more SIDS.
- Any experimental, quasi-experimental and natural experimental evaluation design.
- Quantitative outcomes reported—impact on any aspect of diet (eg, measures of dietary intake or dietary behaviour, sale or purchase of, or expenditure on food, nutrition knowledge or attitude, feeding practices).
- Interventions implemented since January 2000.
- Publications in any language.

Exclusion criteria

- Multi-setting interventions that do not disaggregate SIDS data.
- No intervention implemented (eg, cross-sectional research design).
- Alcohol consumption as only measure of dietary intake.
- Breastfeeding interventions (excluded at title and abstract screen).
- Interventions implemented before January 2000.

(SIDS, Small Island Developing States).

### Search strategy

A comprehensive search strategy, in 11 databases from health, social and agricultural sciences was developed, piloted and then conducted in July 2020 in 11 databases (see review protocol and online supplemental box 1 for details). Reference lists of included studies were checked for other potentially relevant studies as well as reference lists of identified reviews, including those that included grey literature in their searches.

10.1136/bmjnph-2021-000410.supp1 Supplementary data

The search included all studies published since 1 January 2000. No language restrictions were applied; however, we acknowledge a limitation to our search in that all search terms were written in English and most databases searched were primarily English language based. Where the report provided insufficient detail to assess eligibility, study authors were contacted for further details.

### Study selection

Identified citations were uploaded into the online bibliographic database, Rayyan.^16^ Title and abstracts were screened in duplicate by four pairs of reviewers. When eligibility was in doubt, the full text was reviewed. Discrepancies between pairs of reviewers were resolved by a third reviewer.

### Data extraction

Eligible full texts were extracted in duplicate into an online data extraction form.^17^ Data extracted included that necessary to meet our objectives and assess risk of bias (see review protocol for details). Any discrepancies in the extracted data were resolved by a third reviewer.

### Risk of bias in individual studies

Each included study was evaluated for risk of bias using the Cochrane Risk of Bias Tool for randomised trials and the Cochrane ROBINS-I tool for non-randomised studies.^18–20^ Risk of bias was evaluated for the primary outcome measure of interest, in most cases dietary intake, but where not reported (n=4), risk of bias was assessed on another primary outcome such as nutrition knowledge, or food sale or purchase.

### Synthesis of results

Given the heterogeneity of the studies identified, descriptive or narrative synthesis was used to summarise the results. We were particularly interested to determine if interventions promoted a local food approach, and define any intervention components that could be included as such. We did not limit the search, or bias the selection and management of articles, to those that promoted a local food approach, but attempted to identify any aspect of a local food approach within the included studies.

### Patient and public involvement

The need for and scope of this systematic review has been informed by previous work that involved engagement with stakeholders from across the food systems in Jamaica, St Vincent and the Grenadines, St Kitts and Nevis, and Fiji.^21 22^ The findings of this review will be used in further engagement activities and the co-creation of interventions.

## Results

### Study selection

**Figure 1:**
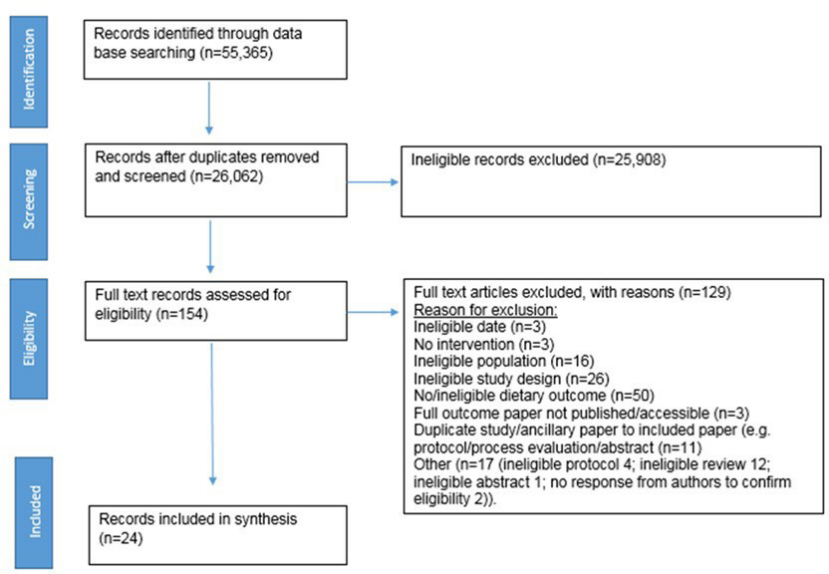
Preferred Reporting Items for Systematic Reviews and Meta-Analyses (PRISMA) flow chart. Flow chart summarising the identification and selection of studies.

Twenty-six thousand unique records were identified, of which 24 were included in the review (figure 1).

### Study characteristics

**Figure 2:**
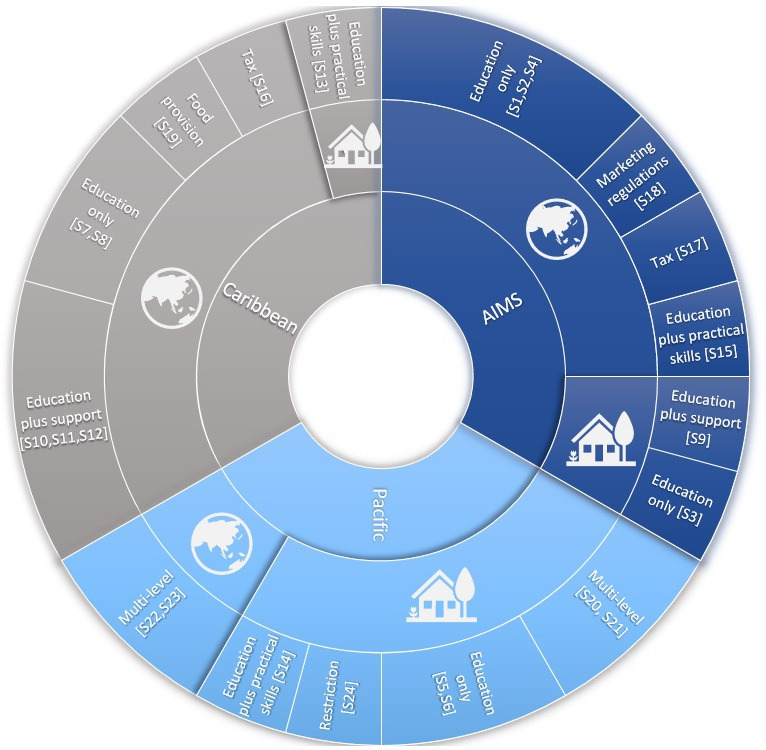
Diet-related interventions in Small Island Developing States summarising study location, focus on local or non-local food approach, and the type of intervention. Sunburst diagram of all 24 included studies showing local food approaches represented with the house icon and non-local approaches represented with the globe icon.

Of the 24 eligible studies, 4 were from separate arms of two multi-country projects.^(S20-S23)^ Figure 2 summarises studies by regional location, focus or not on a local food approach and the type of intervention. Table 1 indicates the country location of the studies, their outcome measures and their risk of bias. Online supplemental table 1 provides a detailed overview of studies that included a focus on local food production, and online supplemental table 2 provides the study characteristics for all 24 included studies.

**Table 1:** Overview of country location, study design, outcome measures and risk of bias for included studies

Ref	Region/country	Study design		Local*	Measured outcome	Evidence for effectiveness†	Risk of bias
	**Nutrition education**						
S1	Singapore	Individually randomised parallel group trial		No	**Purchase**—foods high in sugar	**+/−**	
S2	Singapore	Non-randomised controlled pre/post-test study		No	**Dietary intake**— carbohydrate, protein, vegetable	**+**	
S3	Mauritius	Non-randomised controlled pre/post-test study		Yes	**Dietary intake**— calcium	**+**	
S4	Mauritius	Non-randomised uncontrolled pre/post-test study		No	**Dietary intake**—fruit and vegetable	**+**	
					**Nutrition knowledge** score	**+**	
S5	American Samoa	Non-randomised uncontrolled pre/post-test study		Yes	**Nutrition knowledge** score	**+**	
S6	Guam	Non-randomised uncontrolled pre/post-test study		Yes	**Dietary intake**—fruit and vegetable	–	
					**Willingness to try** fruit and vegetables	–	
S7	Puerto Rico	Non-randomised uncontrolled pre/post-test study		No	**Dietary intake**—daily energy and fibre, fruit and vegetable	–	
S8	Trinidad and Tobago	Cluster-randomised parallel group trial		No	**Dietary intake**—fruit, vegetable, soda, fried food, high fat, salt or sugar food (HFSS).	**+/−**	
					**Nutrition knowledge** score	**+**	
					**Attitudes** to eating	–	
	**Nutrition education plus additional support**						
S9	Singapore	Individually randomised parallel group trial		Yes	**Dietary intake**— energy, carbohydrate, protein, total fat, cholesterol, calcium, dietary fibre, sodium	–	
S10	Puerto Rico	Individually randomised parallel group trial		No	**Dietary intake—** bread, SSB (self-reported)	–	
S11	Trinidad and Tobago	Non-randomised uncontrolled pre/post-test study		No	**Dietary intake**—daily and 7-day fruit and vegetables	**+**	
S12	Trinidad and Tobago	Non-randomised controlled pre/post-test study		No	**Nutrition knowledge** attitude practice score (mean)	**+**	
	**Nutrition education plus practical skills**						
S13	Dominican republic	Non-randomised controlled pre/post-test study		Yes	**Dietary intake**—vitamin A-rich foods	**+/−**	
S14	Federated States of Micronesia	Non-randomised uncontrolled pre/post-test study		Yes	**Dietary intake**—frequency of consumption of various food groups	**+/−**	
S15	Singapore	Non-randomised uncontrolled pre/post-test study		No	**Dietary intake**—whole grains (self-reported)		
					**Nutrition knowledge**—wholegrain specific	**+**	
	**Actual or hypothetical tax**						
S16	Barbados	Interrupted time series study		No	**Sales**—Sugar-sweetened beverages (SSBs).	**+**	
S17	Singapore	Individually randomised multiarm parallel group trial		No	**Purchase** of taxed products	**+**	
	**Advertising/marketing regulations**						
S18	Singapore	Non-randomised uncontrolled pre/post-test study		No	**Dietary intake**—HFSS, fruits and vegetables and nutrient dense foods	**+/−**	
					**Purchase**—snacks (sweets and potato chips, burgers), fruit, vegetables	**+/−**	
	**Food provision**						
S19	Puerto Rico	Non-randomised controlled before-after study		No	**Dietary intake**—various macronutrient and micronutrients	–	
	**Multi-level intervention**						
S20	Tonga	Non-randomised controlled before-after study		Yes	**Dietary intake**—breakfast, fruit, vegetables, SSBs, fruit drink, and various snacks	–	
					**Purchase**—snack food from shop or takeaway after school	**+**	
S21	Fiji	Non-randomised controlled pre/post-test study		Yes	**Dietary intake**—fruit, vegetable, soft drink, fruit drink/cordial	–	
S22	Fiji	Non-randomised uncontrolled pre/post-test study		No	**Dietary intake**—mean population salt intake	–	
S23	Samoa	Non-randomised uncontrolled pre/post-test study		No	**Dietary intake** salt	–	
					**Nutrition knowledge, attitude and behaviour**—salt related	**+/−**	
	**Restriction**						
S24	Solomon Islands	Non-randomised controlled before-after study		Yes	**Dietary intake—**energy, protein, fat	**+**	

Region: Pacific, Caribbean, AIMS.

ROB: low risk, moderate risk, high risk, severe/critical risk.

*Local defined as included process or outcome related components as defined in table 2.

†+ mostly significantly effective on outcomes measured; ± some significant positive effects plus some no change/insignificant effect; − no significant positive effects or negative effect.

AIMS, Atlantic, Indian Ocean, Mediterranean and South China Sea; ROB, risk of bias.

**Table 2:** Overview of aspects of ‘local’ approach within included studies (n=9)

Study reference number			S5	S6	S24	S3	S13	S20	S14	S21	S9
**Aspects of local**	Process related		Afele Fa Amuli 2009	Aflague 2019	Aswani 2007	Bhurosy 2013	Binford 2012	Fotu 2011	Hanson 2011	Kremer 2011	Li 2019
		**Priority setting and intervention development:** Drawing on local expertise and involvement in co-developing intervention objectives and tools (eg, local organisations).									
		**Recruitment**: Drawing on local expertise and involvement in recruiting participants/communities (eg, community headmen/leaders).									
		**Implementation**: By local organisations (eg, Non-government organisations (NGOs)) or individuals rather than a research team from outside the SIDS.									
		**Evaluation**: Drawing on local expertise and involvement in data collection and/or analysis.									
	**Outcome related**	**Promote locally produced food**									
		**Promote traditional/cultural dietary behaviours:** Such as traditional cooking techniques or culturally valued foods.									
		**Consider food composition relevant to local food:** Through nutrient analyses.									

SIDS, Small Island Developing States.

### Location and design of selected studies

Eight studies were conducted in the Pacific region; one each in American Samoa,^(S5)^ Guam,^(S6)^ Federated States of Micronesia (FSM),^(S14)^ Samoa,^(S23)^ Solomon Islands^(S24)^ and Tonga, ^(S20)^ and two in Fiji.^(S21, S22)^ Eight were conducted in the Caribbean; one in Barbados,^(S16)^ one in Dominican Republic^(S13)^, three in Puerto Rico^(S7, S10, S19)^ and three in Trinidad and Tobago.^(S8, S11, S12)^ Eight were based in the Atlantic, Indian Ocean, Mediterranean and South China Sea (AIMS region); two in Mauritius^(S3, S4)^ and six in Singapore.^(S1, S2, S9, S15, S17, S18)^

Nineteen of the studies employed a non-randomised study design; 10 uncontrolled pre/post-test, ^(S23, S5, S6, S14, S7, S11, S4, S2, S15, S18)^ 8 controlled pre/post-test ^(S21, S20, S24, S13, S19, S12, S3)^ and 1 interrupted time series study.^(S16)^ Five were randomised study designs; three individually randomised parallel group trials,^(S10, S1, S9)^ one individually randomised multi-arm parallel group trial^(S17)^ and one cluster-randomised parallel group trial.^(S8)^

### Types of interventions

A large proportion of interventions focused on nutrition education (n=15) and included education that targeted adults in the community, ^(S3, S4, S5, S11, S13, S14)^ clinic ^(S12, S9, S15)^ or workplace setting,^(S2)^ children at school^(S6, S7, S8)^ or young adults at university,^(S10)^ and four targeted women only. ^(S4, S9, S11, S13)^ Three of the education interventions included a skill acquisition component, such as gardening or cooking workshops,^(S13, S14, S15)^ and four provided additional support through individual face-to-face sessions,^(S11)^ mindfulness lessons to promote healthy diets,^(S10)^ food coaching from an app^(S9)^ or counselling from a dietitian.^(S12)^ Two interventions implemented a tax (one real-world 10% levy on sugar-sweetened beverages^(S16)^ and one hypothetical tax on high-calorie products via an online choice experiment.^(S17)^ An online, hypothetical choice experiment evaluated different types of front-of-pack nutrition labelling.^(S1)^ Four interventions were multifaceted, including education, social marketing, community gardens and advocacy for change among manufacturers and retailers; two of these were implemented at population level^(S22, S23)^ and the other two targeted secondary school children.^(S20, S21)^ The final four interventions implemented strategies for building capacity for improved diets in a variety of ways (healthy school meals,^(S19)^ a portion guidance plate for hospital staff,^(S2)^ targeted advertising and marketing of unhealthy foods to children^(S18)^ and marine protected areas to protect community fish stocks.^(S24)^ Six of the interventions included a physical activity component.^(S5, S8, S10, S21)^

### Local food approaches and their effectiveness

Figure 2 provides a visual summary of the frequency of ‘local food’ versus non-local food approaches applied across the 24 studies. A narrative overview of the effectiveness of all 24 studies is available in online supplemental box 2.

### Local food approach to outcome-related components

Nine^(S21, S20, S5, S6, S14, S24, S13, S3, S9)^ of the 24 studies included some form of local approach (table 2). Eight of these nine studies included the promotion of locally produced food or traditional dietary behaviours such as traditional cooking techniques or foods of cultural significance.^(S3, S5, S6, S13, S14, S20, S24)^ Further details on these studies are documented in online supplemental table 1. The other study did not promote local food specifically but examined the nutritional composition of local food in their measure of nutrient intake.^(S9)^ Five of these nine interventions included a practical food production component, such as teaching skills for own food production.^(S6, S13, S14, S21, S20)^

Of the eight studies that specifically promoted locally produced food, four showed significant improvements in dietary intake and one study that did not measure dietary intake showed significant improvement in nutrition knowledge. Of those that improved dietary intake, two were garden-based nutrition education interventions, supplemented with practical skills components (one in Dominican Republic and one in FSM), an education-only intervention targeting adults in Mauritius, and a study that implemented marine protected areas in the Solomon Islands to improve food security for local communities.^(S24)^ The intervention that improved nutrition knowledge involved culturally appropriate strategies to educate adults in American Samoa.^(S5)^

Three studies included a practical local food production component but were not shown to improve diet. These included a summer camp-based intervention in Guam^(S6)^ and two separate arms of the regional Pacific Obesity Prevention in Communities Project in Fiji^(S21)^ and Tonga.^(S22)^

### Local food approach to process-related components

All but one^(S9)^ of the nine studies applied a local approach to process-related components. This included involving local communities,^(S24)^ non-government organisations^(S14, S13)^ or national government^(S23)^ in the prioritisation or development phase of the intervention, employing strategies to enhance the cultural appropriateness of the intervention (such as piloting evaluation tools with members of the community,^(S21, S13)^ translating intervention resources or evaluation tools into the most commonly used language)^(S20, S5, S14, S3)^ or recruiting local facilitators, such as local non-government organisations to deliver culturally appropriate education or implement the intervention.^(S5, S14)^

### Non-local food approaches and their effectiveness (n=15)

The findings from the non-local studies showed mixed effectiveness on dietary intake. Only two showed consistent evidence for improving dietary intake, both using lessons and educational materials to teach adults about nutrition.^(S4, S15)^ Four studies demonstrated mixed effectiveness across measures of intake and six were ineffective.^(S7, S9, S10, S11, S22, S23)^ The three studies that measured impact of tax or labelling interventions on purchasing or sales of unhealthy products were effective.^(S1, S16, S17)^ For details of all study findings, see online supplemental box 2.

### Risk of bias

Table 1 presents the overall risk of bias for the individual studies and online supplemental figure 1 presents the risk of bias for each domain of the tools used.^18 19^ Overall, 13% (n=3) of studies had low risk, 50% (n=12) moderate or some concern, 33% (n=8) high or serious risk and 4% (n=1) had critical risk, that is, unable to provide any useful evidence on the effects of intervention.^18 19^ All eight studies that specifically promoted the consumption of locally produced food were either moderate or high risk of bias.^(S3, S24, S13, S14, S20, S6, S21)^ All were non-randomised studies and the main sources of risk were for confounding and missing data in study reports.

## Discussion

This systematic review identified 24 studies of interventions in SIDS that aimed to improve diet. Variation in study objectives, measured outcomes and quality makes it difficult to draw generalisable conclusions on the effectiveness of these interventions, or to conclude whether incorporating a local food approach into interventions made them more or less effective than their non-local equivalents. However, as there is a particular interest in SIDS in strengthening local, sustainable food systems to address drivers of malnutrition,^1 2^ a comparison of those which focused on a local food approach, versus those that did not, provides interesting learnings which have potential implications for future work.

In placing the findings of this review in the wider context of what is required for change, it is important to consider how SIDS food systems function and where interventions could promote a shift in how people source their food, the type of food they source and consume, and their diet-related health. One example of research in SIDS that has taken a food systems perspective used group model building methodology with local stakeholders to represent food systems as causal loop diagrams in Jamaica, St Vincent and the Grenadines, and St Kitts and Nevis.^22^ These illustrate potential coordinated intervention points to improve diet in these settings; examples include trade agreements and policies that favour imports, impacts on (and opportunity for government to improve) capacity for local agricultural production through knowledge, skills and resilience to shocks, improved access to land for food production and strengthened local supply chains, the relative availability, price and advertising of unhealthy food, cultural norms and social acceptability. This work indicates what is required to enhance local food production for local consumption in SIDS and emphasises that change is required across the food system, beyond a shift in individuals’ knowledge and attitude.

This breadth of potential levels of intervention was not reflected in the research identified by this review. The most implemented type of intervention across the included studies was education (63% (n=15)). Although effectiveness of these interventions varied, those that supplemented education with practical skills training (such as vegetable gardening or cooking demonstrations) showed greatest promise with improvement in dietary intake across all three studies, two taking a local food approach. This is consistent with the findings of educational interventions conducted in other settings, whereby those with additional components such as cooking classes or gardening are more likely to be effective.^23^ However, most of the reviewed studies provide little information as to why their interventions may have worked or not and for whom. The authors of one local education intervention in FSM present findings from an accompanying qualitative study.^(S14)^ They highlight the perceived desirability of imported alternatives over local food (in the Pacific) and emphasise the importance of challenging these perceptions. This requires understanding of the wider sociocultural context, including the food traditions and values attached to different food sources and food types.^24–27^ Local food interventions may be easier to implement in some SIDS populations where traditional foods and methods are highly valued in the context of health than in others where a strong preference for modern, westernised dietary patterns is well established.^28^

The effective and culturally ‘strong’, nutrition education and practical skills interventions in this review^(S13, S14)^ support the notion that cultural relevance, combined with improvements in nutrition knowledge, has the potential to act as a mechanism to dietary change in these settings.^24^ Frameworks that help to identify various ways that cultural relevance can be encompassed within intervention designs may be integral to their effectiveness in these settings.^29^ Local food interventions may need to go beyond the nutrition-education, or skills-based components, and invite communities to explore sociocultural meanings of food and co-develop strategies accordingly.

### How can local approaches contribute to effectiveness?

The findings from this review suggest that local approaches may add a level of contextual relevance to support local food production and the consumption of that food in SIDS that is missing from non-local interventions. This is particularly evident within education and skills interventions that are relatively low-cost and feasible in these resource-limited settings, and already being implemented by local non-government organisations. Such approaches have the potential to reshape perceptions, at individual level, around food preferences which are largely driven by strong sociocultural factors, and therefore should be encouraged as part of future interventions to increase demand for local, healthy foods. However, higher level government intervention is also required, and research into how local governance structures can be strengthened to prioritise local produce over corporate and import markets has been identified as important in SIDS settings.^30^ One example of these higher level infrastructural changes is realised in ‘farm to fork’ programmes that enable nutrition-sensitive value chains to improve local production capacity, and increase availability of healthy, local foods; an approach which may be appropriate in SIDS settings.^1^

For SIDS and similar settings, cross-sectional data suggest that the way food is sourced may impact the diversity of diets^12^ and that own production of a range of crops may contribute to greater dietary diversity.^13^ In the context of disproportionate impacts of climate change and the COVID-19 pandemic on SIDS, there is an urgent need for high-quality interventional research to explore novel ways to implement and evaluate local approaches to ensure food security and sovereignty for the future.^1^ The heterogeneity across studies included in this review preclude quantitative data synthesis and limit a comparison of effectiveness across interventions. This highlights that, in order to do so, we require a standard universal tool for evaluating food production diversity and that allows us to analyse the association between food sourcing, what people are producing themselves, and dietary diversity in order to better understand the impacts of improving local food production on diet and health.

### Reflections on defining local food

Our aim to identify and classify interventions that took a local food approach was challenging to realise, both in terms of clearly defining what we meant by ‘local food’ (table 2) and interpreting, from the published reports, how study authors defined the term. In the studies included, local food was usually implicitly defined by concepts of tradition and cultural value^(S6, S14)^ and/or whether it was locally (geographically) grown or produced.^(S3, S5, S6, S14, S24)^ However, we acknowledge the multitude of ways that ‘local food’ could be defined, such as the felt ‘sense’ of locality that varies between individuals,^31^ or other ways in which food is sourced including community-level sharing, borrowing, exchanging or in a way that is perceived as ‘more local’ such as a small ‘local’ shop or food market that may not necessarily provide food that is produced nearby. As it remains unclear what ‘local food’ means in a given context,^32^ a standard definition or typology is required to produce comparable evidence on the impacts of local food production and consumption versus other approaches. One example of such is that produced for this review, which is presented in table 2.

Understanding the nutritional implications of consuming local over imported food is important, especially for SIDS that are disproportionately impacted by the globalised food system, its drivers and consequences.^2 33^ One study emphasised how traditional and cultural food practices create opportunities for healthy dietary behaviours (such as fruit and vegetable consumption) for many indigenous cultures,^(S6)^ but it is important to acknowledge that ‘local’ may not necessarily mean ‘healthy’, and in this context it is important that definitions of local food, explicitly refer to minimally processed, seasonal and nutritious food.^14^

We considered whether including local versus non-local stakeholders to intervention components impacted effectiveness. However, while acknowledging potential value engaging local stakeholders, there were too few studies describing intervention development to draw generalisable conclusions. In addition, it is possible that the long-term effectiveness of these programmes is enhanced by such engagement. Thus, the true value of ‘local approach’ interventions may not be fully portrayed by the relatively short follow-up in most of the studies we found.

### Limitations

Our review is subject to a general limitation of nutrition-related research, that is, by the accuracy and reliability of the dietary assessment methods, particularly issues obtaining valid dietary recall data using subjective, retrospective methods.^34 35^ As noted in the previous section, a lack of a standard typology for local food is a further limitation. It is imperative to understand *how* these interventions are effective, and the inclusion criteria for this review were not designed to find accompanying qualitative studies.

## Conclusion

It is crucial that we understand the effectiveness of interventions that aim to improve diet in SIDS to inform future strategies to reduce the very high burdens of poor nutrition. This review provides a summary of the evidence on the impact of interventions on aspects of diet and indicates that there appears to be potential for promoting effectiveness by applying a local food approach, through mechanisms of cultural and contextual relevance. However, standard tools, indices and definitions, are required to increase the comparability of these studies.

Additional references can be found in online supplemental file 2.

10.1136/bmjnph-2021-000410.supp2 Supplementary data

## Data Availability

All data relevant to the study are included in the article or uploaded as supplementary information.
