# Supplementary material for: Interventions in Small Island Developing States to improve diet, with a focus on the consumption of local, nutritious foods: a systematic review
Source: BMJ Nutr Prev Health. 2022 Oct 20;5(2):243–53. doi: 10.1136/bmjnph-2021-000410 (PMC9813623; doi:10.1136/bmjnph-2021-000410)
Supplement: Supplementary data [file bmjnph-2021-000410supp001.pdf]

Supplementary Information File

Contents

Supplementary Table 1 - Overview of studies that included a focus on local food production.

Supplementary Table 2 - Study characteristics for all included studies.

Supplementary Figure 1 - Risk of bias for individual studies.

Supplementary Box 1 – Search terms: Medline

Supplementary Box 2 - Narrative overview of effectiveness of local and non-local food approaches

Supplementary Table 1: Overview of studies that included a focus on local food production.

Study	Title	Aim	Study design	Type of intervention	Outcomes/Tools	Effectiveness on measured outcomes	Lessons/takeaways
Afele-Fa'amuli 2009 (S5)	Effectiveness of a Pilot Community Physical Activity and Nutrition Intervention in American Samoa	To assess the effectiveness of a pilot community physical activity and nutrition intervention in American Samoa	Non-randomised controlled before -after	**Nutrition education** – culturally appropriate strategies	**Nutrition knowledge score**/ **Nutrition survey**	**Nutrition knowledge** –increase in knowledge. Almost twice as many participants were able to identify correctly ≤50% of the high-fat foods. 68.2%. More participants were able to identify high fibre foods between base line and follow up.	Emphasises how important it is to develop culturally-appropriate/sensitive programs for effectiveness and ownership.
Aflague 2019 (S6)	Examining the Influence of Cultural Immersion on Willingness to Try Fruits and Vegetables among Children in Guam: The Traditions Pilot Study	To assess the effectiveness of 'cultural immersion' on willingness to try fruits and vegetables among children in Guam.	Non-randomised uncontrolled before -after	**Culturally adapted nutrition lessons** – planting/gardening local produce using traditional and modern practices.	**Dietary intake** (fruit and vegetables) and **willingness to try** fruit and vegetables/ **Adapted WillTry Tool**	**Dietary intake and willingness to try** - No significant change in willingness to try, or difference in post-FV intakes between the camps after adjusting for potential confounders in regression model.	Using existing community programmes to implement new interventions is a feasible approach to research in resource-limited environments.<br>Future research involving cultural immersion should include methods and instruments that can help to capture the cultural context and impact of cultural exposure.
Aswani 2007 (S24)	Do Marine Protected Areas Affect Human Nutrition and Health? A Comparison between Villages in Roviana, Solomon Islands	To assess whether MPAs influenced local perceptions of governance, environmental change, livelihood strategies, and actual human nutrition and health.	Non-randomised controlled before-after study	Marine protected areas	**Dietary intake** - energy, protein, fat/ **24 hour recalls** and **food consumption survey.**	**Dietary intake** - members of villages with effective MPAs had higher energy and protein intakes (particularly marine-derived protein) than those that did not have MPAs or had ineffective ones. Poorest dietary protein/fat ratio was found in the village with the worst MPA governance (attributed to high dependency on cash that subsistence economy for livelihood security and to availability of cash rather than effective marine governance and management.	
Bhurosy 2013 (S3)	Effectiveness of a Theory-Driven Nutritional Education Program in Improving Calcium Intake among Older Mauritian Adults	To improve consumption of calcium-rich foods among older adults through a nutrition education program and gain a better understanding of	Non-randomised controlled before-after study	Education materials and sessions for increasing calcium intake	**Dietary intake** (calcium)/ **Food frequency questionnaire** - 35 item coded self-administered questionnaire.	**Dietary intake** - statistically significant higher mean calcium frequency scores post-intervention ($p < 0.001$).	Suggest that using a theory based (health belief model) nutrition education program and focusing on consumption of calcium rich foods (food-based approach) over calcium supplementation contributed to the success of the program and

		how sociodemographic factors predict calcium intake in this target group.					demonstrates feasibility of the approach among older Mauritian population.
Binford 2012 (S13)	A Garden-Based Nutrition Intervention In The Rural Dominican Republic - Impact On Vitamin A Rich Food Consumption And Household Food Security	To characterize the effect of a garden-based nutrition intervention on vitamin A food frequency and food security measures	Non-randomised controlled before -after	Garden based nutrition education, patio vegetable gardens, and laying hens	**Dietary intake** - vitamin A rich foods/ **Food frequency questionnaire**	**Dietary intake** - children in intervention communities averaged 48.3 servings of vitamin A rich foods/week compared to 44.0 servings/week in control communities. The increase in the all communities' vitamin A food frequency scores was attributed primarily to the large increase in mango and milk consumption. The consumption of garden-specific vitamin A rich foods (green leafy vegetables, carrots, beats) was greater in intervention than communities.	Implicitly emphasises the importance of pilot testing questions regarding food sources, food security and food consumption (e.g. serving size etc.) to ensure that data collected is accurate and in a suitable format for data analysis and interpretation of findings.
Fotu 2011 (S20)	Outcome results for the Ma'alahi Youth Project, a Tongan community-based obesity prevention programme for adolescents	To assess the effectiveness of Ma'alahi Youth Project; a community based intervention for the prevention of obesity in adolescents in Tonga	Non-randomised controlled before -after	Multifaceted intervention - community capacity building, social marketing, education and activities promoting physical activity and local fruit and vegetables	**Dietary intake** – frequency of breakfast, lunch from home. Servings/day fruit, vegetables, SSBs, fruit drink, and various snacks. **Purchase** - snack food from shop or takeaway after school/ **Questionnaires** – knowledge, behaviour and attitudes.	**Dietary intake** - no consistent evidence for improved dietary intake in the intervention group. Some evidence that aspects of diet worsened compared to the control group; SSB intake (OR (adj) 1.69 (p=0.005)), snacking after school (2.21 (0.001)), eating takeaway or fried foods after school (2.76(0.001)), breakfast consumption (0.63 (0.02)). However, reported purchasing of snack food from shop or take away reduced (OR 0.55 (p 0.001)).	Important to account for strong impact of socio-cultural factors on behaviours, particularly for populations where socio-cultural factors are strong determinants of eating, physical activity and body size perceptions.
Hanson 2011 (S14)	An Evaluation of a Nutrition Intervention in Kapinga Village on Pohnpei, Federated States of Micronesia	To encourage return to a more traditional diet to prevent disease, through growing local food, cooking classes, container gardening, and charcoal oven workshops.	Non-randomised uncontrolled before -after	Community-based promotion of container growing, charcoal oven cooking through workshops and classes	**Dietary intake** - frequency of consumption of staples (local starch food, rice, flour products), fruit and vegetables (local vegetables, local fruits, imported fruits, imported vegetables), protein (local fish and seafood, local meat, imported meat, eggs, imported fish and seafood), high fat foods (turkey tail, fried	**Dietary intake** – significant increase in local fruits consumption (1.2 to 2.9 (p<0.001) mean days/week), local vegetables consumption (2.8 to 4.6 (p<0.001), and imported vegetables (0.7 to 2.0 (p<0.001). Local fish and seafood consumption increased from 2.5 to 4.4 (p<0.001) mean days/week with no significant change in imported sources. There was a significant increase in local and imported meat sources (p 0.004; 0.03) and increase in both local and imported sources of starch/flour products, however the local source was not a significant increase (p 0.17; 0.06). There was also a significant increase in	Focus on more familiar and less sensitive traditional crops already planted in the village. Emphasise the importance of education and supervision as component of this type of intervention.

					foods), beverages and snacks (imported drinks with sugar, imported sugar foods, imported salty foods, local snack foods)/ **Food frequency questionnaire -** based on the Helen Keller International 7-day FFQ but modified for use in the FSM and further for Pohnpei.	imported sugary drinks and foods (p 0.011; 0.04).	
Kremer (S21)	Reducing unhealthy weight gain in Fijian adolescents: results of the Healthy Youth Healthy Communities study	To address the issue of adolescent obesity in Fiji.	Non-randomised controlled before-after study	Range of individual to school policy level interventions. Nutrition education focused on reducing energy dense and sugary food/drink, healthy food provision in schools. Included PA component.	**Dietary intake** - Fruit, vegetable, soft drink, fruit drink/cordial. **Questionnaire** -self-administered electronically.	**Dietary intake** - proportion consuming no vegetables/day increased in the intervention group (from 44% to 48%) compared to control group.	The sociocultural studies within the Pacific OPIC project reported that physical, economic and sociocultural forces are especially important determinants of adolescent dietary and activity habits and consequently point to a need for more comprehensive strategies to influence cultural values and expectations.

Supplementary Table 2: Study characteristics for all included studies.

Study ref	Study design	Local food promoted	Other local componenet	Study authors	Region/Country	Population/ subgroup studied	Sample size (loss to follow up)	Intervention	Control	Measured outcomes*	Tools	Effectiveness on outcomes of interest (to this review)
	**Nutrition education**											
S1	Individually randomised parallel group trial	No	No	Ang 2019	AIMS/ Singapore	Adults	512 (0%)	Front of package labelling/warning	Less information/detailed labelling.	**Purchase** - foods high in sugar.	**Online hypothetical grocery store**	**Purchase** - a text-only warning label generated a statistically significant reduction in labelled products purchased. None of the secondary outcomes (total sugar purchased (g), sugar purchased per dollar spent (g per $), total spending ($) and total expenditure on high-in-sugar products ($)) were statistically different across groups.)
S2	Non-randomised controlled pre/post-test study	No	No	deKorn 2017	AIMS/ Singapore	Hospital staff	IG:266 CG:299 (not repeat measures)	Plate with portion guidance.	Normal plate.	**Dietary intake** – carbohydrate, protein, vegetable.	**Plate** with recommended proportions displayed.	**Dietary intake** –after 6 months of the design plate, guideline adherence had significantly increased for vegetables and carbohydrates.
S3	Non-randomised controlled before-after study	Yes	Yes	Bhurosy 2013	AIMS/ Mauritius	Adults over 40 years	IG:98 (0%) CG:91 (0%)	Education materials and sessions for increasing calcium intake.	Education materials only.	**Dietary intake** - calcium	**Food frequency questionnaire** - 35 item coded self-administered questionnaire	**Dietary intake** - statistically significant higher mean calcium frequency scores post-intervention (p <0.001).
S4	Non-randomised uncontrolled pre/post-test study	No	No	Cannoosamy 2016	AIMS/ Mauritius	Housewives	200 (0%)	Nutrition education – lectures and educational materials.	Pre-intervention	**Dietary intake** - fruit and vegetable. **Nutrition knowledge** score.	**FFQ –** specifically fruit and vegetables.	**Dietary intake** – significant increase in number of servings of fruit and vegetable (mean change +0.26 (p < 0.001). **Nutrition knowledge** - significant increase in the nutrition knowledge score (mean change +17.1, (p < 0.001)
S5	Non-randomised uncontrolled pre/post-test study	Yes	Yes	Afele Fa Amuli 2009	Pacific/ American Samoa	Adults	95 (0%)	Nutrition education – culturally appropriate strategies (also PA component)	Pre-intervention	**Nutrition knowledge** score	**Nutrition survey**	**Nutrition knowledge** –increase in knowledge. Almost twice as many participants were able to identify correctly ≤50% of the high-fat foods. 68.2%. More participants were able to identify high fibre foods between base line and follow up.

S6	Non-randomised uncontrolled pre/post-test study	Yes	Yes	Aflague 2019	Pacific/ Guam	3-12 year old summer camp children	105 (6%)	Culturally adapted nutrition lessons	Pre-intervention	**Dietary intake** - fruit and vegetable.<br><br>**Willingness to try** fruit and vegetables.	**Adapted WillTry Tool**	**Dietary intake and willingness to try** - No significant change in willingness to try, or difference in post-FV intakes between the camps after adjusting for potential confounders in regression model.
S7	Non-randomised uncontrolled pre/post test study	No	No	Pinto 2014	Caribbean/ Puerto Rico	School children (8th grade)	32 (16%)	Nutrition education (also PA component).	Pre-intervention	**Dietary intake** – daily energy and fibre, fruit and vegetable.	**24 hour dietary recall –** three times.	**Dietary intake** - no significant changes between baseline and post intervention kcal intake, fruit, vegetable or fibre consumption.
S8	Cluster-randomised parallel-group trial	No	No	Francis 2010	Caribbean/ Trinidad and Tobago	Primary school children (10-11 years)	IG:281 (12%) CG:299 (25%) TG:579 (18%)	Nutrition lessons on the six food groups, sources of nutrients and types of food (also PA component).	No intervention.	**Dietary intake** – servings/day and in last 24 hours of; fruit, vegetable, soda, fried food, HFSS.<br><br>**Nutrition knowledge** score. **Attitudes** to eating.	**Modified block FFQ** with resources including display pictures and actual serving sizes of cook and raw vegetables, beverages, juices and fruits.<br><br>**'Basic nutrition test'** (developed for study).<br><br>**Children's Eating Attitude Test-26 (ChEAT26).**	**Dietary intake** - intervention was not significantly associated with fruit and vegetable intakes. In multivariate regression equations controlling for age, gender, BMI and baseline value, intervention was significantly associated with lower intake levels of fried foods, sodas, HFSS foods.<br><br>**Nutrition knowledge** – in multivariate regression equations controlling for age, gender, BMI and baseline value, intervention was significantly associated with higher knowledge scores.<br><br>**Attitude** - intervention was not significantly associated with ChEAT26 score.
	**Nutrition education plus additional support**											
S9	Individually-randomised parallel-group trial	No	Yes	Li 2019	AIMS/ Singapore	Overweight/ obese pregnant women - clinic	IG:15 (20%) CG:15 (7%) TG:30 (13%)	Food coaching app that provides support and guidance on healthy choices.	Standard educational materials on basic nutrition principles and recommended gestational weight gain.	**Dietary intake** - energy, carbohydrate, protein, total fat, cholesterol, calcium, dietary fiber, sodium	**24-hour dietary recall** – 3 x covering 2 weekdays, one weekend day.<br><br>**Self-administered 3-day food diary.**	**Dietary intake** - no significant differences in changes between control and intervention groups.

S10	Individually-randomised parallel-group trial	No	No	Halperin 2018	Caribbean/ Puerto Rico	University/college students (18-19 years)	IG:20 (10%) CG:21 (0%) TG:41 (5%)	Support sessions focusing on stress reduction and mindfulness approach to diet and PA changes.	Standard care – provision of basic educational materials on diet and PA.	**Dietary intake** – bread, SSB (self-reported).	**Food frequency questionnaire** (multicultural).	**Dietary intake** - insignificant reduction in consumption of soda or bread between the control and intervention group.
S11	Non-randomised uncontrolled pre/post test study	No	No	White 2006	Caribbean/ Trinidad and Tobago	Women (40-60 years)	Pre:42 Post:44 (not repeat measures)	Health-focused support meetings to promote fruit and veg consumption (also promoted PA and cervical screening).	Pre-intervention	**Dietary intake** - daily and 7 day fruit and vegetables.	**Food frequency questionnaire** - based on previous 7 days.	**Dietary intake** – reduction in proportion consuming >5 and >4 servings/day (pretest: 26.19% consuming >5 serving/day; 45.24% >4 servings per day. At 6 months, 6.98% > 5 /d; 18.61% > 4/d)
S12	Non-randomised controlled before-after study	No	No	Webb 2016	Caribbean/ Trinidad and Tobago	Individuals with Type 2 Diabetes – clinic	IG:86 (0%) CG:36 (0%) TG:122 (0%)	Nutrition counselling from Registered Dietitian.	No counselling.	**Nutrition knowledge** score (mean).<br><br>**Attitude** score (mean)<br>**Practice** score (mean)	**27-item KAP structured questionnaire** – for nutrition knowledge.	**Practice** - patients receiving nutrition counselling were more likely than those not receiving counselling to not drink soft drinks (p < 0.001), consume fast foods (p < 0.001) and drink alcohol (p = 0.003) but were equally likely to drink at least eight glasses of water daily.
	**Nutrition education plus practical skills**											
S13	Non-randomised controlled before-after study	Yes	Yes	Binford 2012	Caribbean/ Dominican republic	Pregnant women and children	43 (42%)	Nutrition education, patio vegetable gardens, and laying hens – building self-efficacy by providing vit A rich foods.	No intervention.	**Dietary intake** - vitamin A rich foods	**Food frequency questionnaire.**	**Dietary intake** - children in intervention communities averaged 48.3 servings of vitamin A rich foods/week compared to 44.0 servings/week in control communities. The increase in the all communities' vitamin A food frequency scores was attributed primarily to the large increase in mango and milk consumption. The consumption of garden-specific vitamin A rich foods (green leafy vegetables, carrots, beats) was greater in intervention than communities.
S14	Non-randomised uncontrolled	Yes	Yes	Hanson 2011	Pacific/ Federated States of Micronesia	Adults	75 (10%)	Workshops on container gardening, charcoal oven	Pre-intervention.	**Dietary intake** - frequency of consumption of staples (local	**Food frequency questionnaire -** based on the Helen Keller	**Dietary intake** – significant increase in local fruits consumption (1.2 to 2.9 (p<0.001) mean days/week),

	pre/post test study							use and recipe demonstrations.		starch food, rice, flour products), fruit and vegetables (local vegetables, local fruits, imported fruits, imported vegetables), protein (local fish and seafood, local meat, imported meat, eggs, imported fish and seafood), high fat foods (turkey tail, fried foods), beverages and snacks (imported drinks with sugar, imported sugar foods, imported salty foods, local snack foods)	International 7-day FFQ but modified for use in the FSM and further for Pohnpei.	local vegetables consumption (2.8 to 4.6 (p<0.001), and imported vegetables (0.7 to 2.0 (p<0.001). Local fish and seafood consumption increased from 2.5 to 4.4 (p<0.001) mean days/week with no significant change in imported sources. There was a significant increase in local and imported meat sources (p 0.004; 0.03) and increase in both local and imported sources of starch/flour products, however the local source was not a significant increase (p 0.17; 0.06). There was also a significant increase in imported sugary drinks and foods (p 0.011; 0.04).
S15	Non-randomised uncontrolled pre/post test study	No	No	Goh 2017	AIMS/ Singapore	Patients and carers - clinic	Unclear.	Nutrition education including cooking demonstrations and food samples to try at home.	Pre-intervention.	**Dietary intake** - whole grains (self-reported).<br><br>**Nutrition knowledge** – wholegrain specific	**Questionnaire** – administered via telephone interview	**Dietary intake** - self reported increase in the frequency of consumption and purchasing of whole grains (84% of participants reported positive changes in their dietary habits.).<br><br>**Nutrition knowledge** – statistically significant increase in knowledge score.
	**Actual or hypothetical tax**											
S16	Interrupted time series study	No	No	Alvarado 2019	Caribbean/ Barbados	Whole population (Barbados)		10% taxes on sugar sweetened beverages (SSBs)	No tax	**Sales** - SSBs (weekly volume in millitres sold per capita for SSBs and non-SSBs, carbonated- SSBs, other SSBs, water and other non-SSBs).	**Electronic point-of-sale data**	**Sales** - 10% tax was associated with a 4.3% (95%CI 3.6 to 4.9%) decrease in grocery store sales of SSBs and 5.2% (95%CI 4.5 to 5.9%) increase in sales of non-SSBs.

S17	Individually-randomised multi-arm parallel-group trial	No	No	Doble 2020	AIMS/ Singapore	Adults	IG1:271 (20%) IG2: 298 (14%) IG3: 282 (20%) CG: 293 (17%) TG:1144 (18%)	Hypothetical tax on high calorie products (online)	No hypothetical tax (online).	**Purchase** – proportion of taxed products, kcal per serving, kcal/$, total spend, taxed produced in $	**Online hypothetical grocery store** - NUSMart.	**Purchase** - statistically significant 3.35 percentage point decrease in purchases (95 % CI -6.01 to -0.5) in the explicit tax arm compared to control. Insignificant changes in implicit tax arm compared to control (0.08, 95 % CI -3.31 to 1.77) or in the fake tax arm compared to the control (2.59, 95 % CI -5.04 to 0.00).
	**Advertising/marketing regulations**										
S18	Non-randomised uncontrolled pre/post test study	No	No	Lwin 2020	AIMS/ Singapore	School children (10-17 years)	2818 (0%)	Reduced children's exposure to marketing and advertising of energy dense nutrient poor food and drink.	No information – pre intervention.	**Dietary intake** - sugary foods, high sodium and fat food in comparison to consumption of fruits and vegetables and nutrient dense foods.<br><br>**Purchase** - snacks (sweets and potato chips, burgers) fruit, vegetables.	**Food consumption survey** – self reported<br><br>**Home food inventory checklist** (56 items)	**Dietary intake** - significant reduction in self-reported consumption of potato chips and candies post-implementation compared to pre-implementation. Mean(SD) scores pre and post: Chips 1.97(0.72) to 1.91(0.69); Candies 2.18(0.89) to 1.92(0.85) (note scores 1= consume none to 4= consume every day). No sig change in fruit, veg or burger.<br><br>**Purchase** – household stock of unhealthy convenience foods (selected processed foods) reduced (from mean(SD) 721.65(806.94) to 526.16(736.11) p=0.001). No significant difference in amount of fruit/vegeatables.
	**Food provision**										
S19	Non-randomised controlled before-after study	No	No	Preston 2013	Caribbean/ Puerto Rico	School children (5th, 8th, 1th grade)	IG:189 (0%) CG:132 (0%) TG:321 (0%)	Free healthy school meals.	No free school meals.	**Dietary intake** – energy (total calories, % protein, % carbohydrate, % fat and % saturated fat), vitamins (A, B1, B2, B3, B6, B9, B12, C, D, E),	**24-hour recall. –** three to four in one week.<br><br>**Minnesota Nutrient Data System 32** – to analyse nutrient intake from recalls.	**Dietary intake** –generally few significant improvements in diet over on –participants. Non-participants had a significantly lower energy (kcal) intake than participants (mean(SD) 2177 ± 728 vs 2378 ± 792). No significant difference in energy requirements or macronutrients (% EAR or AMDR for total fat,

										minerals (calcium, iron, magnesium, phosphorous, potassium, zinc) and cholesterol, fiber, sodium, trans-fatty acids.		sat fat, trans fat, carbohydrate, protein). % consuming < EAR was significantly lower in the participant group for six out of 17 vitamins and minerals, when adjusted for energy intake; Vitamin A, B1, Folate, Iron, Magnesium, Zinc, and mean % AI for calcium was significantly greater in participants when adjusted. Authors considered study didn't address high intake of fat and sat fat or sodium which is concern.
	**Multi-level intervention**											
S20	Non-randomised controlled before-after study	Yes	Yes	Fotu 2011	Pacific/ Tonga	Secondary school children (11 to 19 years)	IG:1083 (25%) CG:1396 (36%)	Complex multifaceted intervention (included capacity building, social marketing, community vegetable garden and planting fruit trees).	No intervention.	**Dietary intake** – frequency of breakfast, lunch from home. Servings/day fruit, vegetables, SSBs, fruit drink, and various snacks.<br><br>**Purchase** - snack food from shop or takeaway after school.	**Questionnaires** – knowledge, behaviour and attitudes.	**Dietary intake** - no consistent evidence for improved dietary intake in the intervention group. Some evidence that aspects of diet worsened compared to the control group; SSB intake (OR (adj) 1.69 (p=0.005)), snacking after school (2.21 (0.001)), eating takeaway or fried foods after school (2.76(0.001)), breakfast consumption (0.63 (0.02)). However, reported purchasing of snack food from shop or take away reduced (OR 0.55 (p 0.001)).
S21	Non-randomised controlled before-after study	Yes	Yes	Kremer 2011	Pacific/ Fiji	Secondary school children (13-18 years)	IG:879 (67%) CG:2069 (55%)	Range of individual to school policy level interventions. Nutrition education focused on reducing energy dense and sugary food/drink, healthy food provision in schools. Included PA component.	No intervention.	**Dietary intake** - Fruit, vegetable, soft drink, fruit drink/cordial.	**Questionnaire** - self-administered electronically.	**Dietary intake** - proportion consuming no vegetables/day increased in the intervention group (from 44% to 48%) compared to control group.

S22	Non-randomised uncontrolled pre/post test study	No	No	Pillay 2017	Pacific/ Fiji	Adults	Pre:169 Post:272 (not repeat measures)	Range of interventions at individual to policy level to promote salt reduction. Targeted food producers, retailers, consumers – education to reformulation of products.	Pre-intervention.	**Dietary intake** - mean population salt intake	**24 hour urine samples**<br><br>**Food frequency questionnaire** - stated in protocol but not mentioned in this paper.	**Dietary intake** – no significant reduction in mean salt intake from 24-h urine samples (11.7 grams per day (g/d) at baseline and 10.3 g/d after 20 months (difference: -1.4 g/day, 95% CI -3.1 to 0.3, p = 0.115). Statistically significant reduction in female salt intake in the Central Division (-3.34 (-6.07 to -0.61); p = 0.017) but no differential impact in relation to age or ethnicity.
S23	Non-randomised uncontrolled pre/post test study	No	No	Trieu 2017	Pacific/ Samoa	Adults	479 (39%)	Community level increasing awareness through media and advocacy to reduce salt.	Pre-intervention	**Dietary intake** salt.<br><br>**Nutrition knowledge, attitude and behaviour** - salt related.	**24 hour urinary salt excretion**<br><br>**STEPS survey**	**Dietary intake** - no significant difference in mean salt intake (P=0.588) as measured by 24-h urinary excretion after raking the samples and adjusting for potential confounders.<br><br>**Nutrition knowledge attitude and behaviour** - significant increase in the proportion of participants who understood that high salt consumption could cause serious health problems (from 81 to 90%, P=0.049); decrease in always or often add salt to food when eating (from 50 to 33%, P=0.002) and always or often ate processed foods (from 60 to 49%, P=0.020); increase in using one or more methods to control salt intake (from 73 to 93%, P<0.001), particularly through the use of spices rather than salt in cooking (from 48 to 76%, P<0.001); no change in the proportion who knew the recommended daily salt intake was less than 5 g (22 vs. 20%, P=0.638) or who thought that they consumed too much salt and that lowering salt was important in their diet (P>0.183 for each).; reductions in the proportion of the population who

												reported controlling their salt intake through either checking the sodium on packaged food labels (from 43 to 29%, P=0.015) or avoiding eating out (from 62 to 40%, P<0.001).
	**Restriction**											
S24	Non-randomised controlled before-after study	Yes	Yes	Aswani 2007	Pacific/ Solomon Island	Adults	IG:519 CG:55	Marine protected areas	No marine protected area	**Dietary intake** - energy, protein, fat.	**Dietary recalls –** every hour (between 7am-8pm) for one week.<br><br>Food consumption **survey.**	**Dietary intake** - members of villages with effective MPAs had higher energy and protein intakes (particularly marine-derived protein) than those that did not have MPAs or had ineffective ones. Poorest dietary protein/fat ratio was found in the village with the worst MPA governance (attributed to high dependency on cash that subsistence economy for livelihood security and to availability of cash rather than effective marine governance and management.

Supplementary Figure 1: Risk of bias for individual studies.

Randomised studies (Cochrane ROB 2)	Bias for randomisation	Bias for deviation from intended intervention	Bias for missing data	Bias for measurement of outcomes	Bias in selection of reported result	Overall ROB
Ang 2019	green	green	green	green	green	green
Doble 2020	green	green	yellow	green	yellow	yellow
Francis 2010	green	yellow	red	yellow	green	red
Halperin 2018	yellow	green	green	green	yellow	yellow
Li 2019	yellow	green	yellow	green	yellow	yellow

Non-randomised studies (Cochrane ROBINS-I)	Bias for confounding	Bias for selection of participants into the study	Bias for classification of interventions	Bias for deviation from intended intervention	Bias for missing data	Bias for measurement of outcomes	Bias in selection of reported result	Overall ROB
Afele-Faamuli 2009*	yellow	green	green	green	yellow	yellow	green	yellow
Aflague 2019*	yellow	green	yellow	yellow	yellow	yellow	yellow	yellow
Alvarado 2019	green	green	green	green	green	green	green	green
Aswani 2007*	green	green	yellow	green	green	green	yellow	yellow
Bhurosy 2013*	yellow	yellow	yellow	yellow	yellow	yellow	yellow	yellow
Binford 2012*	red	red	yellow	red	red	yellow	red	red
Cannoosamy 2016	yellow	yellow	yellow	yellow	yellow	yellow	yellow	yellow
deKorn 2017	yellow	yellow	yellow	yellow	yellow	yellow	yellow	yellow
Fotu 2011*	yellow	green	green	green	yellow	yellow	green	yellow
Goh 2017	red	green	green	green	red	yellow	green	red
Hanson 2011*	red	green	green	green	yellow	yellow	green	red
Kremer2011*	yellow	green	green	green	red	yellow	green	red
Lwin 2020	yellow	yellow	yellow	yellow	yellow	yellow	green	yellow
Pillay 2017	yellow	yellow	green	green	red	green	green	red
Pinto 2014	yellow	green	green	green	green	yellow	green	yellow
Preston 2013	green	green	green	green	green	green	green	green
Trieu 2017	green	green	green	green	red	green	green	red
Webb 2016	blue	blue	green	green	red	red	green	blue
White2006	red	yellow	green	yellow	red	yellow	green	red

Key: green = low risk, yellow = moderate/some concern risk, red = high/serious risk, blue = critical risk.
*indicates the studies which applied a local food approach.

Supplementary Box 1: Search terms for Medline

Search identification number	Search terms	Results
1	(Anguilla.tw) OR (Antigua.tw) OR (Antilles.tw) OR (Aruba.tw) OR (Bahamas.tw) OR (Barbuda.tw) OR (Barbados.tw) OR (Belize.tw) OR (Bermuda.tw) OR (Caicos.tw) OR (Caledonia.tw) OR (Caribbean.tw) OR (Cayman.tw) OR (Comoros.tw) OR (“Cook Islands”.tw) OR (Cuba.tw) OR (Curacao.tw) OR (Dominica.tw) OR (Dominican.tw) OR (Fiji.tw) OR (Grenada.tw) OR (Grenadines.tw) OR (Guadeloupe.tw) OR (Guam.tw) OR (Haiti.tw) OR (Jamaica.tw) OR (Kiribati.tw) OR (Lesotho.tw) OR (“Saint Lucia”.tw) OR (“St. Lucia”.tw) OR (Maarten.tw) OR (Madagascar.tw) OR (Maldives.tw) OR (Marshall.tw) OR (Martinique.tw) OR (Mauritius.tw) OR (Melanesia.tw) OR (Micronesia.tw) OR (Montserrat.tw) OR (Nauru.tw) OR (Nevis.tw) OR (Niue.tw) OR (Pacific.tw) OR (Palau.tw) OR (Papua.tw) OR (Polynesia.tw) OR (Principe .tw) OR (Kitts.tw) OR (Samoa.tw) OR (“Sao Tomé”.tw) OR (Seychelles.tw) OR (Singapore.tw) OR (“small island developing states” .tw) OR (Solomon.tw) OR (Suriname.tw) OR (Timor-Leste.tw) OR (Tonga.tw) OR (Trinidad.tw) OR (Tobago.tw) OR (Tokelau.tw) OR (Turks.tw) OR (Tuvalu.tw) OR (“Puerto Rico”.tw) OR (Marianas.tw) OR (Martinique.tw) OR (Vanuatu.tw) OR (Verde.tw) OR (“Saint Vincent”.tw) OR (“St. Vincent”.tw) OR (“Virgin Islands”.tw)	113648
2<br>(these terms base on keywords from GBD dietary risk factors and synonyms for SSB used in another review on SSB tax (Backhoeler 2019)	Diet/ OR diet*.tw OR Nutrients/ OR nutrient.tw OR Food/ OR food.tw. OR Nutrition Surveys/ or Nutrition Assessment/ OR nutrition*.tw OR Sugar-Sweetened Beverages/ OR "sugar sweetened beverage$".tw OR SSB.tw OR Fruit/ OR fruit*.tw OR Vegetables/ OR vegetable*.tw OR Micronutrients/OR micronutrient.tw OR macronutrient.tw OR “dietary diversity”.tw OR Whole Grains/ OR wholegrain*.tw OR Dietary Fiber/ OR fibre.tw OR legume*.tw OR pulses.tw OR nut.tw OR seed.tw OR milk.tw OR Red Meat/ OR red meat.tw OR processed meat.tw OR calcium.tw OR Fatty Acids, Omega-3/ OR Carbonated Beverages/ or carbonated beverage.tw OR soda*.tw OR Beverages/ OR Energy Drinks/ OR energy drink*.tw OR soft	3012651

	drink*.tw OR Seafood/ OR fish.tw OR seafood.tw OR Sodium, Dietary/ OR sodium.tw OR salt.tw	
3	assessment.tw OR intervention*.tw OR evaluation.tw OR experiment*.tw OR program*.tw OR strateg*.tw OR initiative.tw OR polic*.tw OR project.tw OR scheme.tw OR plan.tw OR task.tw OR method*.tw OR treatment.tw OR tool.tw OR education*.tw OR tax*.tw OR incentiv*.tw OR communit*.tw OR household*.tw	13368517
4	1 and 2 and 3	8775
5	2000:2020. (sa_year)	7422

Supplementary Box 2: Narrative overview of effectiveness of local and non-local food approaches

Effectiveness of local food approach to outcome-related components

All nine studies in Table 2 applied a local approach to outcome-related components. This included promoting locally produced food and traditional dietary behaviours such as traditional cooking techniques or foods of cultural significance (n=8),[S3, S5, S6, S13, S14, S20, S21, S24] or applying locally-relevant tools to outcomes measures. [S9] Five of these interventions included a practical food production component, such as teaching skills for planting and harvesting own produce. [S6, S13, S14, S20, S21]

Of the eight studies that specifically promoted locally produced food, four showed significant improvements in dietary intake and one study that did not measured dietary intake, showed significant improvement in nutrition knowledge. Two of the effective interventions that demonstrated improved dietary intake were garden-based nutrition interventions. One targeted pregnant women and children in rural Dominican Republic to improve consumption of locally produced food rich in Vitamin A. [S13] The intervention provided garden-based nutrition education, vegetable gardens and laying hens and assessed intake using a FFQ, to significantly increase consumption of any vitamin A rich food, and garden specific vitamin A rich foods in intervention than control communities. The second study aimed to encourage the return to traditional diets to prevent disease in Pohnpei (Federated States of Micronesia), through growing local food using container gardening, and classes on cooking and using charcoal ovens. [S14] This mixed-methods study took a unique approach in focusing on food source as part of their context-specific assessment of dietary intake – distinguishing between consumption of local and imported foods, and found significant increases in the consumption of local and imported fruit and vegetables, local and imported meat, local fish and seafood, imported starch/flour, and imported surgery drinks and foods (Supplementary Table 1).

A third effective intervention that used a local approach to improve dietary intake, was the implementation of Marine Protected Areas (MPAs) in the Solomon Islands. [S24] The study aimed to assess the impact of protecting fish stocks on food security for local communities. Authors observed variation in effectiveness of MPAs in protecting fish stocks, but residents in villages with effective MPAs had higher energy and protein intakes (particularly marine-derived) than those that had ineffective or no MPA. Poorest dietary protein/fat ratio was found in the village with the worst MPA governance, and this was attributed to availability of

and high dependency on cash over subsistence for livelihood security, rather than effective marine governance and management.

The final local intervention that was effective in improving dietary intake, was a quasi-experimental study in Mauritius that applied an educational approach to increase consumption of locally produced sources of calcium such as milk, fish and dark green leafy vegetables, amongst adults aged 40+ years.[(S3)]

Three other studies included a practical local food production component, similar to the first two garden-based studies described, but these were not shown to effectively improve diet. One of these studies was conducted in Guam, targeted school-aged children and involved the implementation of culturally adapted nutrition lessons at a summer camp. [(S6)] Local food production was promoted through skill acquisition and taught participants how to grow and cook their own produce using modern and traditional practices whilst emphasising the impacts of imported versus local food. The study used an adapted *WillTry* tool to assess dietary intake and 'willingness to try' various items, but showed no significant change in dietary intake, or willingness to try, post-intervention. [S6)] The other two were separate arms of the regional Pacific Obesity Prevention in Communities Project in Fiji [(S21)] and Tonga, [(S20)] which involved quasi-experimental studies of community and school-based interventions that included some emphasis on local food production and consumption through vegetable gardens or agricultural training, as a way of building capacity for schools and communities to create solutions to food insecurity.[(S20)] Despite these studies providing no evidence for significant effects on dietary intake, the Tongan study did have a significant effect on reducing purchasing of some unhealthy food items, including snack foods from a shop or takeaway. [(S20)]

One of the nine studies did not measure dietary intake but evaluated change in nutrition knowledge as the primary outcome measure. This study implemented culturally-appropriate strategies to educate adults in American Samoa about nutrition, and demonstrated an increase in knowledge about items such as high-fat and high-fibre foods post intervention. [(S5)]

Only one study, which was a randomised controlled trial conducted in Singapore, considered the unique nutritional composition of local food in their outcome measure, by analysing dietary records using an online nutrient analysis software which was derived from locally available foods.[(S9)] The intervention targeted overweight and obese pregnant women with a food coaching app that provided guidance on healthy choices and resulted in no significant changes to macro or micronutrient intakes.[(S9)] Although the intervention itself did not apply a local approach or focus on the consumption of locally produced foods, it is worth noting that this study was the only study to specifically acknowledge nutrient variation according to food source, and apply that to their analysis; however the findings reported in the manuscript are not specifically discussed in the context of this application.

Effectiveness of non-local food approach (n=15)

Twelve of these studies measured the impact of intervention on dietary intake. Two of these 12 studies were significantly effective, four showed some significant improvement on some, but not other, measures of dietary intake, and six showed no evidence to be effective.

The two studies that were effective, applied an education approach to teach adults about nutrition via lessons and educational materials. [(S4, S15)] Both were quasi-experimental studies conducted in the AIMS region, used FFQs to assess dietary intake outcomes and

demonstrated significant improvements in diet. One targeted housewives in Mauritius and significantly increased the number of servings of fruit and vegetables consumed compared to pre-intervention, [S4] and the other targeted patients and carers attending general outpatients clinic in Singapore with nutrition information, cooking demonstrations and provision of healthy food samples. [S15] The evaluation focused on wholegrain consumption and found a significant increase in frequency of consumption and purchasing of wholegrains. Both of these interventions also measured nutrition knowledge and showed significant improvements in knowledge scores post intervention.

The four studies that demonstrated mixed effectiveness included educational intervention, food provision and regulations on advertising and marketing. One study targeted hospital staff in Singapore to assess the impact of a plate displaying portion guidance compared to a normal plate and found increased vegetable and reduced carbohydrate proportion over six months, but no significant change in proportion of protein.[S2] One study evaluated a free school meals intervention in Puerto Rico. [S19] Using 24-hour recall method researchers found a significant improvement in nutrient intake for some (six of the 17) vitamins and minerals, but no significant improvements in macronutrient or sodium intake. A Trinidad-based study that used nutrition education to target fruit and vegetable intake in school-aged children, showed no significant association between intervention and fruit or vegetable intake in multivariable regression, but provided evidence that the intervention was associated with lower intakes of fried food, soda and food high fat salt and sugar, and improvements in nutrition knowledge.[S8] Similar to that study, the final study to show a mixed effect demonstrated significant reductions in the consumption of two out of three unhealthy items measured (chips, sweets, burgers), but no improvements in fruit and vegetable intake. This intervention, targeting children, was conducted in Singapore and implemented a national policy-level restriction on advertising and marketing of energy dense, nutrient poor food and drink to children.[S18] This study also measured the impact of intervention on purchase of specific food items, and provided evidence of a significant reduction in purchasing of selected processed/convenience foods, but no difference in fruit or vegetable purchasing.

Two of the six interventions that were ineffective at changing dietary intake, applied a support/guidance approach to empower participants to improve dietary intake [12, 15,], and one implemented school-based nutrition education. One targeted women aged 40-60 years in Trinidad and Tobago and implemented health-focused support meetings, specifically to promote fruit and vegetable intake, but demonstrated a reduction in fruit and vegetable consumption following the intervention.[S11] One targeted university students, in Puerto Rico, with support sessions that focused on stress reduction and mindfulness to improve diet and physical activity, but showed no significant reduction in consumption of the two variables assess, SSB or bread, between control and intervention groups. [S10] Another intervention that was ineffective, was the implementation of school-based nutrition and physical activity lessons in Puerto Rico that showed no significant effect on fruit, vegetable, fibre or energy intake, post intervention.[S7]

Finally, two community level interventions, one in Fiji and the other in Samoa, applied a range of interventions as part of salt reduction programmes.[S22, S23] The Fiji study involved multilevel intervention from individual to national policy level and targeted stakeholders across the food system from producers (advocating for reformulation) through to consumer education. [S22] In Samoa, a community level media campaign advocated for reducing salt intake. [S23] Both used non-randomised, uncontrolled pre/post-test designs and measured salt intake through 24-hour urinary salt excretion, and reported no significant reduction in mean salt intake. However, the Samoan study also measured nutrition (specifically salt-

related) knowledge, attitude and behaviour and showed some significant, positive effect on these outcomes.[S23]

The other four of the 15 studies that did not focus on locally produced food, did not measure dietary intake, but evaluated, and showed mixed intervention effects on, other outcomes; purchasing, [S1, S17] sales [S16] or nutrition knowledge [16]. Two studies measured purchases from an online hypothetical grocery store and were based in Singapore; [S1,S17] one assessed the impact of a hypothetical tax of high calorie items on purchase and expenditure, [S17] and the other assessed the impact of various types of food labelling (information) on product choice.[S1] Both found statistically significant changes in some of the purchasing behaviours measured. The explicit tax was associated with a decrease in unhealthy food purchases, which was not shown in the implicit or fake tax arm of the study,[S17] and a text-only warning label was associated with a significant reduction in purchase of labelled products; however, there was no difference in total sugar purchased or total expenditure on high-sugar products.[S1]

One study evaluated the impact of a national 10% tax on sugar-sweetened beverages (SSBs) in Barbados and showed a significant decrease in grocery store sales of SSBs, which was primarily driven by a reduction in carbonated SSB sales, and an increase in sales of non-SSB including significant increase in sales of bottled water. [S16]

The final study, conducted in Trinidad and Tobago, measured the impact of a dietitian-led nutrition counselling intervention on nutrition knowledge, and targeted adults with Type 2 Diabetes.[S12] There was no significant difference in total knowledge score when comparing the intervention and control (no counselling) groups, but did demonstrate a significant improvement in attitude and practice scores amongst those who received the intervention compared to the control.
