# Supplementary material for: Interventions in Small Island Developing States to improve diet, with a focus on the consumption of local, nutritious foods: a systematic review
Source: BMJ Nutr Prev Health. 2022 Oct 20;5(2):243–53. doi: 10.1136/bmjnph-2021-000410 (PMC9813623; doi:10.1136/bmjnph-2021-000410)
Supplement: Supplementary data [file bmjnph-2021-000410supp002.pdf]

References for included studies:

S1. Ang FJL, Agrawal S, Finkelstein EA. Pilot randomized controlled trial testing the influence of front-of-pack sugar warning labels on food demand. *BMC Public Health*. **19**(1):164 (2019).

S2. de Korne DF, Malhotra R, Lim WY, Ong C, Sharma A, Tan TK, et al. Effects of a portion design plate on food group guideline adherence among hospital staff. *Journal of nutritional science*. 6:e60.(2017)

S3. Bhurosy T, Jeewon R. Effectiveness of a Theory-Driven Nutritional Education Program in Improving Calcium Intake among Older Mauritian Adults. *The Scientific World Journal*.750128. (2013)

S4. Cannoosamy K, Pem D, Bhagwant S, Jeewon R. Is a Nutrition Education Intervention Associated with a Higher Intake of Fruit and Vegetables and Improved Nutritional Knowledge among Housewives in Mauritius? *Nutrients*. **8**(12):723.(2016)

S5. Salei'a A-Fa, Whitney K, Mark D. Effectiveness of a Pilot Community Physical Activity and Nutrition Intervention in American Samoa. *Californian Journal of Health Promotion.* **7**(1).(2009)

S6. Aflague TF, Leon Guerrero RT, Delormier T, Novotny R, Wilkens LR, Boushey CJ. Examining the Influence of Cultural Immersion on Willingness to Try Fruits and Vegetables among Children in Guam: The Traditions Pilot Study. *Nutrients*. **12**(1). (2019)

S7. Pinto E, Toro B, Vicéns L. Nutrition and Physical Activity Interventions for Childhood Obesity: Lessons Learned. *Ecology of food and nutrition*. **53**(5):503-13.(2014)

S8. Francis M, Nichols SS, Dalrymple N. The effects of a school-based intervention programme on dietary intakes and physical activity among primary-school children in Trinidad and Tobago. *Public health nutrition*.**13** (5):738-47. (2010)

S9. Li LJ, Aris IM, Han WM, Tan KH. A Promising Food-Coaching Intervention Program to Achieve Optimal Gestational Weight Gain in Overweight and Obese Pregnant Women: Pilot Randomized Controlled Trial of a Smartphone App. *JMIR formative research*. **3**(4):e13013. (2019)

S10. Halperin DT, Laux J, LeFranc-García C, Araujo C, Palacios C. Findings From a Randomized Trial of Weight Gain Prevention Among Overweight Puerto Rican Young Adults. *Journal of nutrition education and behavior*. **51**(2):205-16. (2019)

S11. White SC, Agurto I, Araguas N. Promoting healthy behaviors to prevent chronic disease in Panama and Trinidad & Tobago: results of the women as agents of change project. *Journal of community health.* **31** (5):413-29. (2006)

S12. Webb MC, Aguilal JJ. Nutritional Knowledge, Attitude and Practice among Patients with Type 2 Diabetes in North Central Health Authority in Trinidad and Tobago. *The West Indian medical journal.* **65**(1):170-6. (2015)

S13. Binford J, Camp MA, Camp D. A garden-based nutrition intervention in the rural Dominican Republic - impact on vitamin A rich food consumption. *The FASEB Journal*. 26. (2012)

S14. Hanson M, Englberger L, Duncan B, Taren D, Mateak H, Johnson E. An evaluation of a nutrition intervention in Kapinga Village on Pohnpei, Federated States of Micronesia. *Pacific health dialog.* **17**(1):173-84. (2011)

S15. Goh LML, Wong A, Ang G, Tan AS. Effectiveness of nutrition education accompanied by cooking demonstration. *British Food Journal.*119:1052-66. (2017)

S16. Alvarado M, Unwin N, Sharp SJ, Hambleton I, Murphy MM, Samuels TA, et al. Assessing the impact of the Barbados sugar-sweetened beverage tax on beverage sales: an observational study. I*nternational Journal of Behavioral Nutrition and Physical Activity.* **16**(1):13. (2019)

S17. Doble B, Ang Jia Ler F, Finkelstein EA. The effect of implicit and explicit taxes on the purchasing of 'high-in-calorie' products: A randomized controlled trial. *Economics & Human Biology.* **37**:100860. (2020)

S18. Lwin MO, Yee AZH, Lau J, Ng JS, Lam JY, Cayabyab YM, et al. A macro-level assessment of introducing children food advertising restrictions on children's unhealthy food cognitions and behaviors. *International Journal of Advertising.* 39(7):990-1011. (2020)

S19. Preston AM, Venegas H, Rodríguez CA, Vélez-Rodríguez RM. Assessment of the national school lunch program in a subset of schools in San Juan, Puerto Rico: participants vs. non-participants. *Puerto Rico health sciences journal*. **32**(1):25-35. (2013).

S20. Fotu KF, Millar L, Mavoa H, Kremer P, Moodie M, Snowdon W, et al. Outcome results for the Ma'alahi Youth Project, a Tongan community-based obesity prevention programme for adolescents. *Obesity Reviews*. **41**. (2011)

S21. Kremer P, Waqa G, Vanualailai N, Schultz JT, Roberts G, Moodie M, et al. Reducing unhealthy weight gain in Fijian adolescents: results of the Healthy Youth Healthy Communities study. *Obesity Reviews.***12** Suppl 2:29-40. (2011)

S22. Pillay A, Trieu K, Santos JA, Sukhu A, Schultz J, Wate J, et al. Assessment of a Salt Reduction Intervention on Adult Population Salt Intake in Fiji. *Nutrients*. 9(12). (2017)

S23. Trieu K, Ieremia M, Santos J, Neal B, Woodward M, Moodie M, et al. Effects of a nationwide strategy to reduce salt intake in Samoa. *Journal of hypertension.* 36(1):188-98. (2018)

S24. Aswani S, Furusawa T. Do Marine Protected Areas Affect Human Nutrition and Health? A Comparison between Villages in Roviana, Solomon Islands. *Coastal Management*. **35**(5):545-65. (2007)
